# Surgical intervention for lung cancer in patients aged 75 and above: potential associations with increased mortality rates—a single-center observational study

**DOI:** 10.1186/s13019-024-02922-5

**Published:** 2024-07-29

**Authors:** Andrey Kaprin, Oleg Pikin, Andrey Ryabov, Oleg Aleksandrov, Denis Larionov, Airat Garifullin

**Affiliations:** 1 Federal State Budget Institution National Medical Research Radiology Centre of the Ministry of Healthcare of the Russian Federation (FSBI NMRRC), Moscow, Russia; 2 Peoples’ Friendship University of Russia, Moscow, Russia; 3 P. Hertsen Moscow Oncology Research Institute, 3, 2 Botkinskiy Proezd, Moscow, 125284 Russia; 4 National Medical Research Center of Phthisiopulmonology, Moscow, Russia

**Keywords:** Pulmonary surgical procedure, Elderly, Complications, Perioperative period

## Abstract

**Background:**

Lung cancer, which is diagnosed two to three times more frequently in patients over the age of 70, is a leading cause of cancer-specific mortality. Given the elevated risk of morbidity and mortality, surgical intervention may not always be the most appropriate primary treatment option. This study aims to evaluate specific risk factors associated with postoperative morbidity and mortality in elderly patients and to optimize patient selection therefore improving surgical outcomes.

**Patients and methods:**

The study encompassed a cohort of 73 patients aged 75 and above who underwent surgical treatment for non-small cell lung cancer (NSCLC) at the Department of Thoracic Surgery of the P. Hertsen Moscow Oncological Research Institute between 2015 and 2021. All patients underwent preoperative evaluation, including PET/CT staging and functional assessment, carried out by a multidisciplinary team comprising thoracic surgeons, anesthesiologists, and other medical specialists.

**Results:**

The investigation revealed a postoperative mortality rate of 5.5% and a postoperative morbidity incidence of 16.4%, with occurrences of atrial fibrillation in 41.6%, persistent air leak in 33.3%, and pneumonia in 25% of complicated cases. At the one-year follow-up, 88% of patients remained free from relapse, whereas at three years, this rate stood at 66%. During the follow-up period, 16 patients (22%) passed away, with a median survival duration of 44 months. Survival rates at one year, three years, and five years were 71%, 66%, and 35%, respectively. Multivariate analysis disclosed several significant factors predicting a complex postoperative period, including stage IIIb (*p* = 0.023), pN1 (*p* = 0.049), pN2 (*p* = 0.030), and central location (*p* = 0.007). Additionally, overall survival was primarily influenced by a Charlson comorbidity index of 6 (*p* = 0.044), stage Ia2 (*p* = 0.033), and the necessity for thoracotomy (*p* = 0.045).

**Conclusion:**

Each case of lung cancer in patients aged 75 and older necessitates an individualized approach. Given the higher mortality rate relative to younger patients, comprehensive risk assessment and preoperative management of underlying comorbidities are imperative, with the involvement of anesthesiologists, intensive care physicians, cardiologists, and other relevant specialists as needed.

## Introduction

In contemporary society, the steady increase in life expectancy is a prevailing trend, and it is projected that ongoing medical advancements will further contribute to the ageing of the population. By 2050, approximately 17% of the population is anticipated to be over 65 years old [1]. It is noteworthy that lung cancer, the most prevalent and deadliest of all cancers, predominantly affects individuals aged 70 and older [2]. For instance, while an 80-year-old US citizen has an expected life span of nine years, the median survival time for lung cancer patients in this age group is merely 14 months. A series of questions related to age-related variations in tumour biology, the influence of concomitant medical conditions on the tolerability of cancer treatment or surgery, and the potential benefits of stereotactic body radiation therapy (SBRT) in enhancing long-term survival in localised, resectable lung cancer necessitate thorough investigation. Nonetheless, a common tendency persists to exclude elderly patients from research studies or set restrictive age criteria at 65 years [3]. Elderly patients often face exclusion from studies or are denied surgical interventions not only due to diminished functional capabilities but also on account of their age and the perceived risk of complications [4]. Surgical procedures in elderly patients are characterised by an array of risk factors, encompassing age-related physiological changes resulting in declining organ function and reduced reserve capacity, the presence of comorbid medical conditions, polypharmacy, cognitive and functional impairments, nutritional status concerns, insufficient social support, and issues related to caregiver availability [5]. In numerous instances, major lung resections become impractical due to reduced lung and cardiovascular capacity [6]. The conventional methods of preoperative assessment may prove inadequate, necessitating a more comprehensive evaluation that includes exercise tolerance testing, which is sometimes inaccessible. It is well documented that the incidence of complications increases proportionally with age [7]. To optimise patient selection and, consequently, enhance surgical outcomes, it becomes of paramount importance to comprehend the factors that can impact outcomes in this patient demographic.The main objective of this study was to examine the outcomes of surgical procedures in patients over 75 years of age with non-small cell lung cancer (NSCLC) and to identify the main risk factors that contribute to a complicated postoperative course and reduced long-term survival rate. This article adheres to the STROBE reporting checklist.

## Patients and methods

We gathered data on patients aged 75 and above who underwent lung cancer surgery at the Department of Thoracic Surgery of the P. Hertsen Moscow Oncology Research Institute between 2015 and 2021. Inclusion criteria encompassed individuals aged 75 years or older, a confirmed diagnosis of non-small cell lung cancer (NSCLC), and surgeries performed by our clinical team. The choice of setting the age threshold at 75 for elderly lung cancer patients served several important purposes. First, it adheres to the widely accepted medical and demographic definition of the elderly population, as individuals aged 75 and above often experience heightened age-related physiological changes and comorbid conditions, substantially influencing their health and surgical outcomes. Second, employing a specific age threshold, such as 75, ensured a more uniform study group, enhancing the similarity among patients in terms of age-related factors and health profiles. Consequently, this approach bolstered the internal validity of the study by minimising potential confounding variables. The decision to pursue surgery was made collaboratively by a multidisciplinary tumour board for all cases. Patients with small cell lung cancer and those who had undergone exploratory surgery were excluded from the study. Ethical considerations were paramount; thus, the study was conducted in strict adherence to the Declaration of Helsinki, gaining approval from the P. Hertsen Moscow Oncology Research Institute Ethics Committee. Additionally, participating patients provided written consent, and all clinical data were anonymized. The study received approval from the Institutional Review Board (#034-B, 10.02.2022). Exclusion criteria encompassed patients with incomplete data, those who underwent diagnostic surgical procedures such as mediastinoscopy, and individuals who declined participation in the study.

### Preoperative assessment

Perioperative data encompassed a range of factors, including age, clinical and pathological stage according to the 8th TNM classification, preoperative forced expiratory volume in the first second (FEV1, %), Charlson's Comorbidity Index (CCI) score, date of operation, histology details, radicality of resection, resection margins, growth pattern, extent of surgery, duration, blood loss, type of incision, and bronchial resection. Additionally, postoperative complications were meticulously documented. Patients were categorised into two groups based on the presence or absence of complications. An initial univariate analysis was followed by a multivariate model to elucidate the risk factors associated with adverse outcomes and long-term survival.

Preoperative assessments consisted of an electrocardiogram (ECG), echocardiogram, spirometry test, and venous Doppler ultrasound. Patients with emphysema and respiratory volume depletion underwent assessments of diffusing capacity of the lung for carbon monoxide and VO2max. Individuals with a reduced ejection fraction received further evaluation, involving a Holter ECG monitor and an exercise stress test.

For staging purposes, 18-FDG PET/CT scans were conducted in conjunction with brain MRI. All patients were staged in accordance with the TNM 8th edition. Central tumours with an endobronchial component necessitated endoscopic biopsy, while peripherally located lesions were suspected of lung cancer based on dynamic tumour enlargement and increased PET/CT uptake. In such cases, wedge resection or segmentectomy with a frozen section was performed to rule out benign lesions or tuberculosis. Decisions regarding the extension of parenchymal resection and the performance of mediastinal lymphadenectomy were contingent upon pathological findings. Indications for thoracotomy included central tumours involving the main or lobar bronchi, mediastinal lymphadenopathy, or an inability to tolerate single-lung ventilation. The selection of candidates for surgery followed international guidelines, with preference for surgery in case of atelectasis and tumour disintegration.

Following anatomical pulmonary resection, patients were admitted to the intensive care unit (ICU) for 24 h. This observation period was reduced to 2 h after wedge resection or segmentectomy. Removal of the pleural drain occurred once the outflow volume diminished to 300 ml within 24 h, provided that the fluid exhibited no chylous or blood characteristics, and air leakage had ceased. All patients underwent rehabilitation following the fast-track protocol employed in our department. This protocol encompassed preoperative patient education, thoracic epidural anaesthesia, early extubation, and the encouragement of early mobilisation. In cases of persistent fluid accumulation, a series of repeat thoracenteses were performed. The decision for discharge was made when the patient was fully rehabilitated and no longer required analgesics or antimicrobial treatment.

Postoperative complications were assessed based on the Clavien‒Dindo classification [8], and the preoperative Charlson Comorbidity Index (CCI) score was documented for all patients [9]. To examine any potential bias in the advantages of minimally invasive surgery, a comparison was made between the preoperative and histological characteristics of patients who underwent thoracotomy and those who underwent video-assisted thoracoscopic surgery (VATS).

### Power analysis

Before initiating the study, a power analysis was conducted to determine the required sample size for the chi-square test. The primary objective was to assess the association between two categorical variables, namely the presence of a postoperative complication and a 30-day lethal outcome. The following parameters were considered in the power analysis. The effect size was estimated based on pilot data. A moderate effect size was anticipated. A significance level (α) of 0.05 was chosen, indicating a 5% probability of Type I error. A power of 0.80 was selected, representing an 80% probability of detecting an association if it exists. The power analysis indicated that a minimum sample size of 32 participants was required to achieve the desired level of statistical power. This sample size was used as the basis for participant recruitment and data collection.

### Statistical analysis

The primary data acquisition, calculation of assessed parameters, systematisation, and data aggregation were executed utilising our proprietary open-source medical information system developed in C#, employing ASP. Net Core, and backed by PostgreSQL.

For quantitative variables adhering to a normal distribution, the mean and standard deviation were reported alongside a 95% confidence interval. In instances where variables deviated from a normal distribution, descriptive statistics included the median with interquartile range. Categorical data are presented as absolute and relative frequencies. Comparisons of frequencies in multifield contingency tables were conducted via Pearson's chi-square test. When assessing three or more groups concerning a quantitative variable without a normal distribution, the Kruskal‒Wallis test was employed, followed by Dunn’s criterion with Holm correction as a post hoc method. In cases involving three or more groups with a quantitative variable exhibiting a normal distribution, a one-way analysis of variance was conducted, accompanied by the Tukey test as a post hoc method, assuming equal variances. Survival time was calculated from the date of surgery to the last follow-up date using the Kaplan‒Meier method. Variations in survival were analysed through log-rank analysis. Missing data were handled based on the missing at random assumptions. A Cox proportional hazard regression model was applied to perform a multivariate analysis of prognostic factors, while a logistic regression model was employed to identify risk factors for postoperative complications. After multivariate regression, we constructed the Receiver Operating Characteristic (ROC) model to analyze the performance of our predictive model. This involved plotting the true positive rate against the false positive rate and calculating the area under the curve (AUC) to assess the model's discriminative ability. In addition to plotting the ROC curve, we identified the optimal threshold that maximizes both sensitivity and specificity. In addition to plotting the ROC curve, we identified the sensitivity and specificity for predicting the lethal outcome in NSCLC patients aged 75 and above. Surgical mortality encompassed all patients who passed away within the initial 30 days post surgery or during the same hospital stay. Statistical analyses were executed using IBM SPSS Statistics v.26 for Windows (IBM Corporation), StatTech v. 2.8.3 (Developer—StatTech LLC, Russia), and GraphPad Prism 9.0 (GraphPad Software, Inc., San Diego, CA).

## Results

**Fig. 1:**
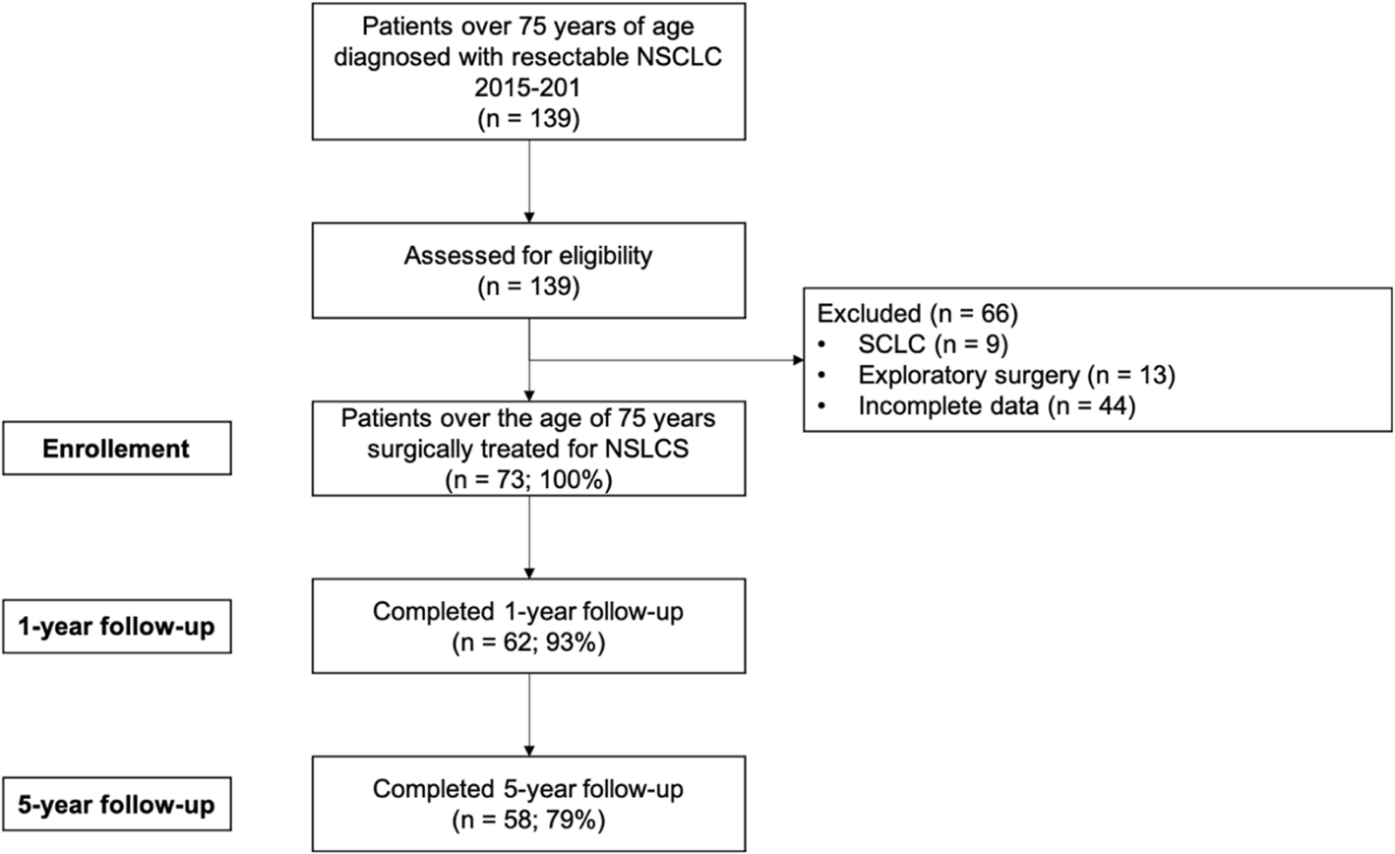
STROBE flow chart. n, number of patients; NSCLC, non-small cell lung cancer; SCLC, small cell lung cancer

The sample size for this study was established after the exclusion of 13 elderly patients who had undergone invasive staging procedures such as mediastinoscopy and parasternal mediastinotomy to assess lymph node involvement, as well as 9 patients diagnosed with small cell lung cancer. These exclusion criteria were implemented to enhance the homogeneity of the patient group under analysis by removing individuals with a history of prior surgical procedures or neoadjuvant treatment. The primary reason for nonparticipation was associated with incomplete data (31.6%). Among the 139 elderly patients who underwent surgery in our department for suspected non-small cell lung cancer (NSCLC), a total of 73 patients were included in the analysis for this study (Fig. 1).

Following the exclusion of patients meeting the criteria, the age of the study participants ranged from 75 to 84 years (median = 78, interquartile range: 77–79), with 45.2% being female and 54.8% male. The decision to proceed with surgery was made collectively by an extended tumor board, including an oncologist, a thoracic surgeon, a radiotherapist, an anesthesiologist, an intensive care physician, and a cardiologist. Out of the participants, 10 (13%) reported complications related to vascular disease, comprising 6 (8%) who had experienced myocardial infarctions and 4 (5%) with a history of cerebrovascular accidents. The Charlson Comorbidity Index (CCI) scores ranged from 5 to 9. Further details regarding baseline patient characteristics are presented in Table 1.

**Table 1:** Baseline patient characteristics

Characteristic	Total *n* = 73
**Age**	
Median, IQR	78, 2
Min—max	75–84
**Sex, n (%)**	
Male	40 (54.8%)
Female	33 (45.2%)
**Stage, n (%)**	
IA2	16 (21.9%)
IA3	13 (17.8%)
IB	9 (12.3%)
IIA	4 (5.5%)
IIB	16 (21.9%)
IIIA	8 11.0%)
IIIB	5 (6.8%)
IVA	2 (2.7%)
**Histology type, n (%)**	
Adenocarcinoma	44 (60.3%)
Squamous cell	23 (31.5%)
Adenosquamous	3 (4.1%)
Small cell	2 (2.7%)
Metastasis	1 (1.4%)
**Adeconarcinoma type, n (%)**	
Acinar	18 (40.9%)
Papillar	7 (15.9%)
Mucinous	5 (11.4%)
Micopapillary	5 (11.4%)
Nonspecified	4 (9.1%)
Lepidic	3 (6.8%)
MIS	1 (2.3%)
Nonmucinous	1 (2.3%)
**pN, n (%)**	
N0	47 (67.1%)
N1	14 (20.0%)
N2	9 (12.9%)
**Growth pattern, n (%)**	
Peripheral	63 (86.3%)
Central	10 (13.7%)
**Type of lung resection, n (%)**	
Lobectomy	50 (68.5%)
Segmentectomy	14 (19.2%)
Pneumonectomy	4 (5.5%)
Bilobectomy	3 (4.1%)
Wedge	2 (2.7%)
**Surgical access, n (%)**	
VATS	38 (52.1%)
Thoracotomy	35 (47.9%)
**Bronchial resection, n (%)**	
Nonsleeve	67 (91.7%)
Sleeve	6 (8.3%)
**Resection margin, n (%)**	
R0	68 (93%)
R1	3 (4%)
R2	2 (3%)

*n *number of patients in each group, *pN *pathologic N, *VATS *Video-assisted thoracic surgery

In our patient cohort, 40 (54%) of the patients exhibited a decline in FEV1 below 90%, a typical age-related occurrence [10]. The mean FEV1 was 88 ± 16%, with a 95% CI of [84, 92]. Notably, the lowest rate (65%) was observed in a long-term smoker with central adenosquamous carcinoma associated with emphysema, while the highest rate (152%) was observed in a patient with peripheral adenocarcinoma devoid of pulmonary comorbidities.

Stage I lung adenocarcinoma was diagnosed in 37 (50.6%) of the patients, with lymph node metastases identified in 25 (32.9%). Among the 9 (12.9%) patients with pN2, preoperative detection of enlarged or hypermetabolic lymph nodes was observed in only 4 (44%) patients, indicating a 7% rate of occult N2 disease. Tumour size ranged from 1 to 14 cm (median = 3.6 cm, interquartile range: 2.1–4.5). In three patients, mediastinal lymphadenectomy was not conducted due to significant comorbidities.

The mean duration of the surgical procedures was 184 ± 58 min, with a 95% CI of [170, 198]. The longest operation, lasting 330 min, was noted during VATS S1 + 2 segmentectomy for adenocarcinoma of the left upper lobe. Blood loss varied from 50 to 750 ml (median = 150 ml, interquartile range: 2.1–4.5). The median duration of chest drainage was 3 days (interquartile range: 2–4). The most prolonged air leakage was observed after a right upper lobectomy with bronchial sleeve resection, followed by a diagnosis of pneumonia in the operated lung that responded to antibiotic treatment. The mean length of hospital stay was 12 ± 2 days, with a 95% CI of [9, 14], and was significantly longer in patients with complications (median = 14.5 days; U = 170, *p* = 0.003) but shorter after VATS (median = 9.5 days; U = 228, *p* < 0.001).

Adjacent organ and structure resection was performed in 15 (20.5%) patients, including sleeve lobectomy in 6 (8.3%), vagus nerve resection in 2 (3%), pericardial resection in 2 (3%), wedge lung resection in 2 (3%), chest wall resection in 2 (3%), and pulmonary artery resection in 1 (1.5%) patient.

Out of the 73 patients, a total of 12 (16.4%) experienced complications, with 4 of them succumbing during their hospital stay, resulting in a 30- and 90-day mortality rate of 5.5%. Notably, the majority of these complications occurred in patients who had undergone thoracotomy, comprising 9 of the 12 cases (75%). The distribution of complications was as follows: 3 (25%) in 2015, 1 (8.3%) in 2016, 1 (8.3%) in 2017, 4 (33.3%) in 2018, 1 (8.3%) in 2019, and 2 (16.6%) in 2021. According to the Clavien‒Dindo classification, grade II complications were diagnosed in 5 (6.8%) patients, and grade IIIa complications were diagnosed in 2 (2.7%) patients. The most prevalent complication was atrial fibrillation, observed in 5 (7%) patients, one of whom succumbed to a massive pulmonary embolism. Persistent air leakage, defined as lasting for more than 5 days, was detected in 4 (5.4%) patients, and in one case, it led to severe pneumonia and death on postoperative day 32. Pneumonia was diagnosed in 3 (4.1%) patients, accounting for 25% of all complications. One patient with hypocoagulation underwent emergency thoracotomy due to hemothorax several hours after the initial operation but eventually succumbed to erosive pulmonary haemorrhage on postoperative day 21. Table 2 summarises the univariate analysis of the risk factors for complications.

**Table 2:** Risk factors for complications

Characteristic	Category	Complication		*p*
		No	Yes	
**Type of lung resection**	Wedge	2 (100,0)	0 (0,0)	0.131
	Bilobectomy	1 (33,3)	2 (66,7)	
	Lobectomy	44 (88,0)	6 (12,0)	
	Pneumonectomy	3 (75,0)	1 (25,0)	
	Segmentectomy	11 (78,6)	3 (21,4)	
**Incision**	Thoracotomy	25 (71,4)	10 (28,6)	**0.007**
	VATS	36 (94,7)	2 (5,3)	
**Bronchial resection**	Non-sleeve	58 (85,3)	10 (14,7)	0.187
	Sleeve	3 (60,0)	2 (40,0)	
**Amount of resection**	Simple	51 (87,9)	7 (12,1)	0.110
	Combined	10 (66,7)	5 (33,3)	
**Stage**	IA2	16 (100,0)	0 (0,0)	**0.036 p**_**IA2 – IIIB**_** = 0.029**
	IA3	9 (75,0)	3 (25,0)	
	IB	9 (100,0)	0 (0,0)	
	IIA	4 (100,0)	0 (0,0)	
	IIB	11 (68,8)	5 (31,2)	
	IIIA	7 (87,5)	1 (12,5)	
	IIIB	2 (40,0)	3 (60,0)	
	IVA	2 (100,0)	0 (0,0)	
	Metastasis	1 (100,0)	0 (0,0)	
**Histologic type**	Adenocarcinoma	41 (93,2)	3 (6,8)	**0.015 p**_**adenocarcinoma– adenosquamous**_** = 0.011**
	Adenosquamous	1 (33,3)	2 (66,7)	
	Small cell	2 (100,0)	0 (0,0)	
	Squamous cell	16 (69,6)	7 (30,4)	
**pN**	N0	44 (93,6)	3 (6,4)	**0.003 p**_**N0 – N1**_** = 0.009** **p**_**N0 – N2**_** = 0.005**
	N1	9 (64,3)	5 (35,7)	
	N2	5 (55,6)	4 (44,4)	
**Growth pattern**	Peripheral	56 (88,9)	7 (11,1)	**0.008**
	Central	5 (50,0)	5 (50,0)	

*p p* value, *pN* pathologic N, *VATS* video-assisted thoracic surgery

**Fig. 2:**
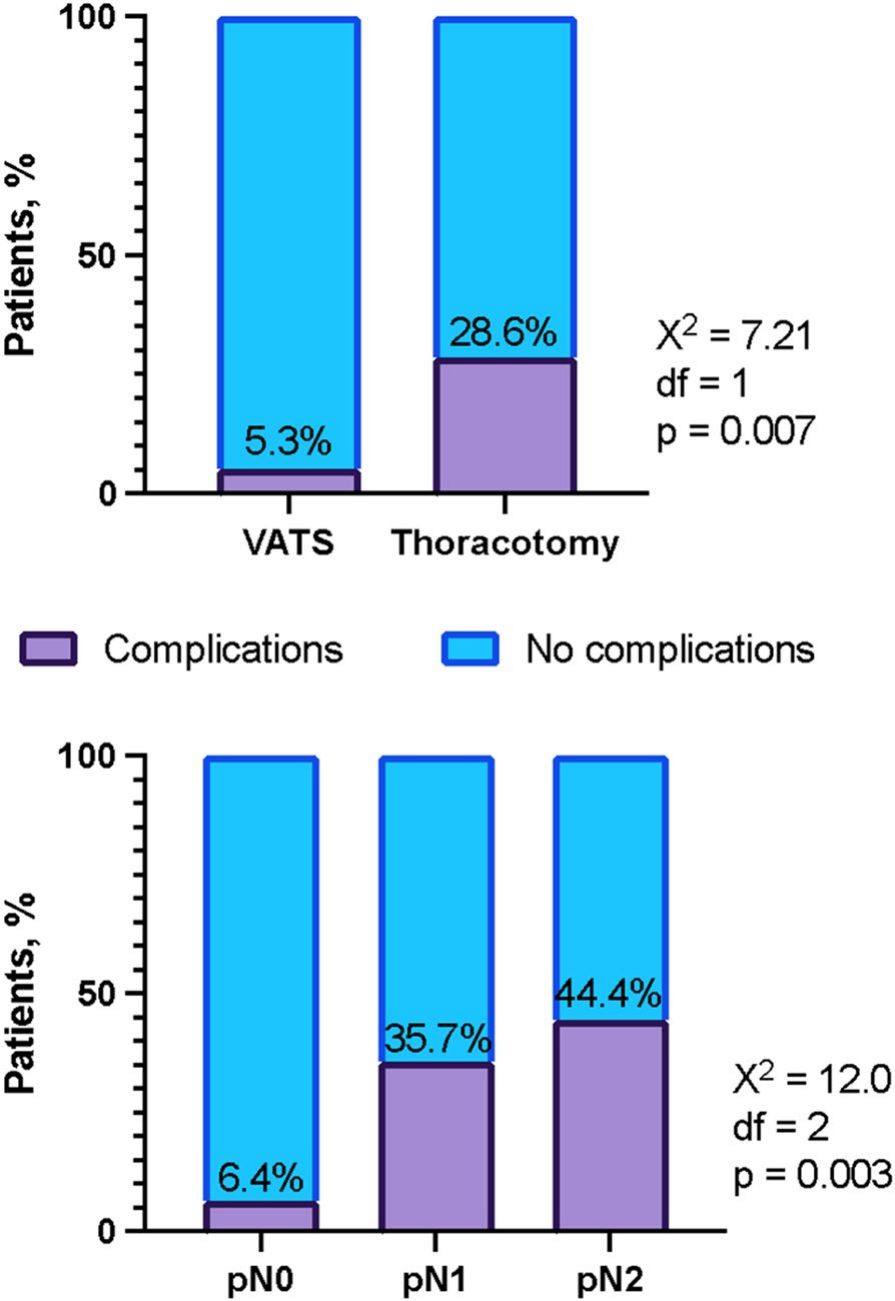
Surgical access, lymph node involvement and complication rate. df, degree of freedom; p, p value; pN, pathologic N; VATS, video-assisted thoracic surgery; X^2^, chi-square

Patients subjected to thoracotomy exhibited a significantly higher incidence of complications than their counterparts who underwent VATS, as demonstrated by a chi-square test (χ2(1) = 7.21, *p* = 0.007) (Fig. 2).

However, it is worth noting that the VATS group exhibited a lower stage (*p* = 0.024), less mediastinal lymph node involvement (*p* = 0.048), fewer combined resections (*p* = 0.001), a higher frequency of lobectomy (*p* = 0.042), and a greater prevalence of adenocarcinoma (*p* < 0.001). Furthermore, the median tumour volume in the thoracotomy group was significantly higher at 3.6 cm than in the VATS group at 2.3 cm (U = 301, *p* < 0.001).

Patients with stage IIIb had a significantly higher complication rate than those with localised stages (χ2(1) = 7.42, *p* = 0.006). Patients with central tumour location were more prone to complications than those with peripheral growth (OR 8.0, 95% CI [1.844, 34.712], *p* = 0.001). Figure 2 also illustrates the increase in complications related to mediastinal lymph node involvement and the type of surgical access.

Quantitative characteristics such as age, duration of surgery, blood loss, preoperative FEV1, tumour size, and CCI score did not show a statistically significant difference between complicated and uncomplicated patients (*p* > 0.005).

**Fig. 3:**
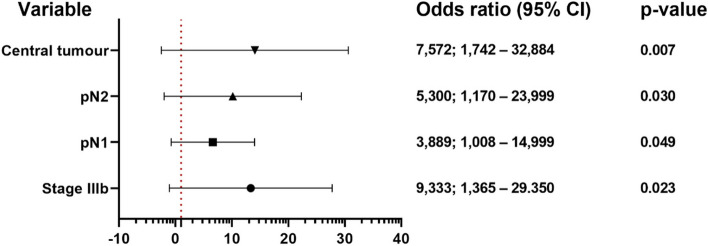
Multivariate analysis of postoperative complications. CI, confidence interval; pN, pathologic N

The multivariate analysis revealed the most significant predictors of a complicated postoperative period (Fig. 3).

The area under the receiver operating characteristic (ROC) curve was 0.930 ± 0.052, 95% CI [0.827, 1], indicating a high capacity of the model to correctly classify positive and negative cases. The model's statistical significance (*p* < 0.001) underscores its strong performance. With a sensitivity of 83.3% and specificity of 82.8%, the model demonstrates a balanced ability to accurately identify true positive and true negative cases, highlighting its clinical utility.

**Fig. 4:**
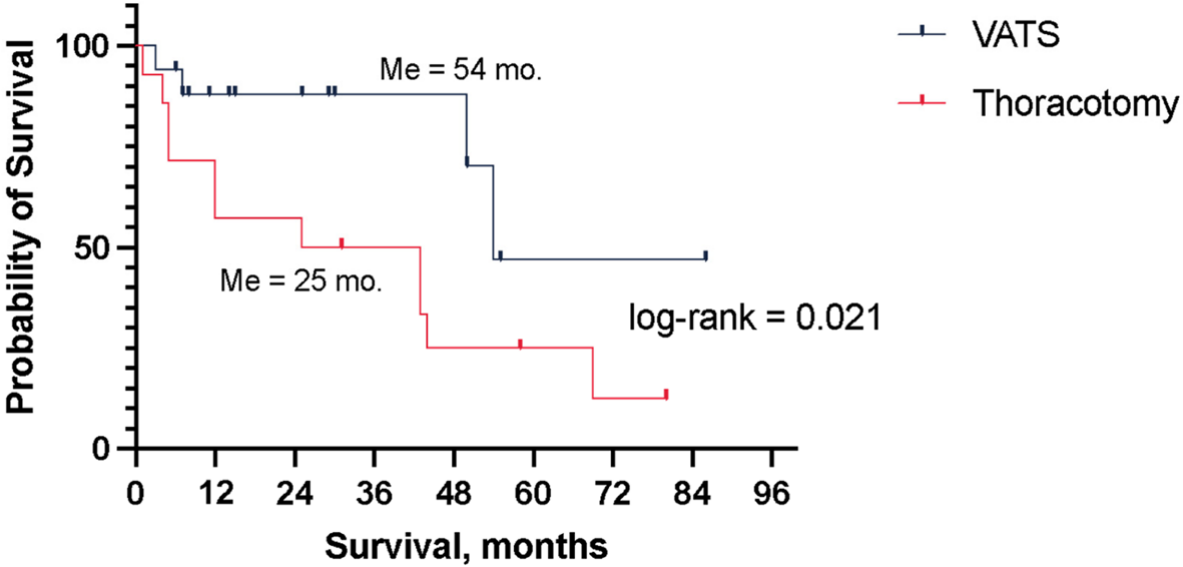
Overall survival stratified by the surgical access method used. Me, overall survival; VATS, video-assisted thoracic surgery

The mean follow-up duration was 25 ± 28 months, 95% CI [20.02, 76.08], ranging from 1 to 82 months. Local relapse occurred in 8 (11%) of the followed patients. At 1 year of follow-up, 88% of patients remained relapse-free (95% CI [0.75, 1]), and at 3 years, this rate was 66% (95% CI [0.42, 0.9]). During the follow-up period, 16 (22%) patients passed away, with a median survival of 44 months (95% CI [25, 69]). The 1-year survival rate was 71% (95% CI [0.54, 0.87]), the 3-year survival rate was 66% (95% CI [0.49, 0.84]), and the 5-year survival rate was 35% (95% CI [0.13, 0.57]). Notably, overall survival after VATS was significantly higher than that after thoracotomy (54 vs. 25 months, *p* = 0.021; Fig. 4).

Multivariable proportional hazards regression analysis revealed that patients with stage I disease and a lower Charlson Comorbidity Index (CCI) experienced better outcomes, while undergoing thoracotomy was the sole significant predictor of poorer outcomes (Table 3).

**Table 3:** Multivariate analysis of overall survival

Predictors	Coefficients	p	HR	95% CI	
				Lower	Upper
**CCI**	1.32	0.044	0.27	0.063	0.864
**Stage Ia2**	-4.18	0.033	0.02	0	0.712
**Stage Ia3**	-4.15	0.034	0.02	0	0.740
**Thoracotomy**	0.49	0.045	1.63	1.010	2.630

*CCI* Charlson Comorbidity Index, *CI* confidence interval, *OR* odds ratio, *p p* value

The area under the receiver operating characteristic (ROC) curve was 0.861 ± 0.068, 95% CI [0.729, 0.994], signifying a reasonably good ability of the model to classify positive and negative cases. The model exhibited statistical significance (*p* < 0.001). With a sensitivity of 75% and specificity of 81.2%, the method displayed a balanced capacity to identify true positive and true negative cases, indicating its clinical utility.

## Discussion

Surgery for lung cancer in elderly patients can be safely conducted at a high-volume cancer centre with substantial experience in both minimally invasive and open surgical techniques. However, it is important to acknowledge that the mortality rate remains notably high in this patient population. A crucial aspect of this process is the meticulous preoperative assessment conducted by a multidisciplinary tumour board, which includes the involvement of anesthesiologists and critical care physicians. The choice between video-assisted thoracoscopic surgery (VATS) and open surgery (thoracotomy) often hinges on patient selection. It is essential to comprehensively evaluate the extent of comorbidities since the long-term outcome for elderly patients with localised lung cancer is strongly influenced by the initial performance level, which can be affected by concurrent medical conditions.

The World Health Organization defines individuals aged 75 or older as elderly [11]. Despite the increasing popularity of stereotactic body radiation therapy (SBRT), surgical intervention continues to be the primary approach for the treatment of stage I-II lung cancer, primarily due to its favourable outcomes concerning local disease control and overall survival [12]. Nonetheless, it is crucial to recognize that surgery in this age group is associated with elevated morbidity and mortality rates [13]. Several studies have reported varying morbidity rates, ranging from 20 to 63%, and mortality rates spanning from 0 to 16% (Table 4).

**Table 4:** Outcomes following surgical treatment of lung cancer in elderly patients

Author	Year	Number of patients	Age	Morbidity, %	Mortality, %
Naunheim [14]	1994	37	> 80	45	16
Berry [5]	2009	338	> 70	47	3.8
Port [15]	2011	121	> 80	63	2.5
Mun [16]	2008	55	> 80	25.6	3.6
Hope [17]	2007	20	> 80	45	10
Okada [18]	2012	44	> 80	20	0
Our study	2023	73	> 75	16.4	5.5

In our study, the postoperative morbidity rate was observed to be 16.4%, a figure that appears slightly lower than the rates reported in previously published series. Several factors may contribute to this disparity. A pivotal element of the fast-track recovery protocol involves the early encouragement of physical activity, which is facilitated by the absence of a chest tube. The prompt removal of the chest tube has been associated with decreased pain levels, a reduced risk of pneumonia, and a lower likelihood of pleural cavity contamination [19]. However, in certain cases where refractory postoperative hydrothorax persists, necessitating repeat thoracentesis, this intervention is typically well tolerated and curative. In our study, we did not categorise these cases as complications, which might have led to a somewhat lower overall morbidity rate compared to other studies. Another contributing factor is the reduced pain levels and infrequent use of opioid analgesics, which likely play a role in reducing the incidence of pneumonia, responsible for only 4% of complications in our study. The investigation also underscored an increased morbidity associated with thoracotomy compared to VATS, emphasising the substantial impact of the surgical approach on patient outcomes. These findings highlight the importance of further exploration and potential refinement of surgical methods, particularly when dealing with elderly patients, to minimise morbidity and optimise patient safety.

It is well known that the overall mortality rate after lung cancer surgery varies between 1.6% and 3.7% [20–23]. However, this rate significantly escalates when operating on older patients, as evident in our study, where it reached 5.5%, and in a study by Naunheim et al., reporting a rate of 16%. The notable increase in mortality among older patients remains a key limiting factor in the decision-making process for surgery, particularly when considering extended resections and dealing with patients with poor functional performance. In our study, four out of twelve patients who experienced complications did not survive, resulting in a mortality rate of 33% among those who encountered postoperative complications.

Atrial fibrillation emerges as one of the most prevalent adverse events during the postoperative period. The incidence of atrial fibrillation is typically low at 0.37–1% after general surgery but can surge to 10–20% after lung parenchyma resection [24, 25]. The rates increase even further following anatomical lung resections, reaching 34.2% after pneumonectomy and 4.5% after lobectomy [26]. Typically, atrial fibrillation is diagnosed on postoperative days 2–3 and has the potential to progress to systemic hypotension, heart failure, and even pulmonary embolism due to the formation of thrombi in atriae. In our study, atrial fibrillation was detected in 42% of complicated cases and in 7% of all enrolled patients.

Regrettably, one patient in our study died of a subsequent massive pulmonary embolism. When dealing with elderly patients, it becomes imperative to conduct a thorough preoperative assessment to rule out any possible cardiac issues and initiate early anticoagulant prophylaxis in high-risk individuals. In cases where significant neurological impairment or cognitive problems arise due to a prior cerebrovascular accident, a comprehensive discussion with the patient's family becomes a necessary step. Additionally, alternative treatment options, such as stereotactic body radiation therapy (SBRT), should be considered, and a judicious decision should be made in such circumstances.

Various tools are available to assess surgical risk, including the NSQIP, SURPASS, Lee, and ASA scoring systems. One of the commonly used tools for evaluating the cumulative severity of comorbidities is the Charlson Comorbidity Index [27]. This index calculates adjusted risk scores based on age and 19 of the most common and significant comorbidities in patients undergoing treatment. It is frequently employed in surgical and chemotherapy planning for a broader oncology population [28]. In our study, the CCI score ranged from a minimum of 5 points, attributed to patients over 70 years old with only the presence of oncology, to a maximum of 10 points, observed in an 83-year-old patient with a history of penile and prostate cancer, emphysema, postinfarction cardiosclerosis, type 2 diabetes, asthma, and an abdominal aortic aneurysm. Given the high surgical risk and the subpleural location in this particular case, we opted to maximise lung parenchyma preservation and expedite the surgery by performing a VATS wedge resection, despite the presence of lung adenocarcinoma. Remarkably, the patient survived for four years without recurrence but eventually succumbed to COVID-19. While univariate analysis revealed that CCI did not predict the complication rate, it was effective in estimating the overall survival of patients in the study. However, patients with a higher number of preoperative complications may undergo a reduced scope of surgical resection, ultimately reducing surgical trauma and the incidence of postoperative complications.

One of the most accurate methods for assessing the functional capacity of a patient scheduled for pulmonary resection is the measurement of VO2max. A higher VO2max generally correlates with better cardiopulmonary fitness and can serve as a valuable prognostic indicator for surgical outcomes [29]. It aids in patient selection, risk stratification, and the development of personalised preoperative optimization strategies, ensuring that individuals with lower VO2max receive appropriate interventions to enhance their functional capacity and mitigate potential postoperative complications. However, VO2max was not incorporated into the current study due to the absence of this facility in the clinic.

The milestone work of Ginsberg and Rubinstein [30] laid the foundation for our understanding of lung cancer resections. Their seminal study demonstrated that lobectomy represents the least radical type of lung resection for lung cancer, and patients who underwent sublobar resection exhibited significantly lower overall and recurrence-free survival rates. While this classic paper, published in 1995, has been widely cited over 640 times, significant advancements in medical sciences, including the advent of PET/CT, new TNM classifications, and VATS, have ushered in substantial changes in the diagnosis, treatment modalities, and comprehension of lung cancer.

Recent studies have challenged the notion that segmentectomy represents a compromise operation, suggesting that it may even be beneficial for lesions less than 2 cm, both in terms of complication rates and long-term survival [31, 32]. Notably, the results of the CALGB 140503 study, which randomised 701 patients with stage IA lung cancer to lobar and sublobar resections, demonstrated that after a 7-year follow-up, sublobar and lobar resections yielded similar disease-free and overall survival rates. The overall survival rates after 5 years were 80.3% for sublobar resection and 78.9% for lobar resection [33].

In our study, segmentectomy was performed in 19.2% of cases, emerging as the second most common procedure following lobectomy (68.5%). Remarkably, the complication rates did not exhibit significant differences between these two groups. Wedge resection has largely faded from the spectrum of treatment options, as it is no longer deemed radical enough and is reserved for severely ill patients, as previously described. Pneumonectomy is also witnessing a decline in popularity due to the poor tolerance of subsequent hemodynamic and ventilatory changes. In cases where pneumonectomy is being considered, it is essential to thoroughly evaluate the feasibility of sleeve resection, a procedure that necessitates a two-stage approach: first during preoperative planning and subsequently during intraoperative assessment. The open sleeve lobectomy technique should be integrated into the standard practice of every thoracic surgery department that manages lung cancer patients.

The introduction of VATS for anatomic lung resection in the early 1990s significantly transformed the landscape of lung cancer surgery. VATS has demonstrated safety, reduced morbidity, and equivalent efficacy when compared to traditional open surgery. This minimally invasive technique has been embraced as the standard surgical approach for early-stage lung cancer and is increasingly employed in more advanced cases, including in elderly patients [34, 35].

In our study, the VATS group exhibited fewer complications than the open approach group (5.3% vs. 28.6%; *p* = 0.007). However, it is important to consider that this discrepancy may partly result from selection bias. VATS procedures were predominantly performed on peripheral small adenocarcinomas without mediastinal lymph node involvement. In contrast, meticulous lymphadenectomy during thoracotomy in the presence of N1 and N2 disease might account for potential damage to vagal parasympathetic nerve branches, leading to a higher incidence of atrial fibrillation in this group [36]. Nonetheless, VATS demonstrated superior results to the open approach, regardless of the stage, and was more favourable for elderly patients due to enhanced postoperative recovery and reduced pain levels. The multivariate analysis underscored that thoracotomy was an independent negative predictor of overall survival.

The 8th edition of the TNM classification, introduced by the Union for International Cancer Control in 2017, brought several significant updates [37]. One of the notable changes was the reclassification of T3N2 patients as stage IIIb, rendering them nonsurgical candidates. Our study included three stage IIIb patients who underwent surgery before the implementation of the new TNM classification. In some cases, surgery may still be considered for advanced-stage patients with the intention of performing salvage procedures for conditions such as abscess, empyema, or massive pulmonary haemorrhage. However, such cases are associated with a heightened complication rate (60% in our study), larger tumour sizes (average of 8 cm), and more extensive lung resections (pneumonectomy or bilobectomy performed in 100% of these cases). Furthermore, complications in these situations carried a 100% fatality rate. Therefore, decisions to operate under such circumstances should be made with the primary goal of saving the patient's life, and the associated risks must be carefully weighed against potential benefits. Nevertheless, in our series, the number of deaths in patients with IIIb and N2 was very low and insufficient for an accurate analysis.

This study exhibits certain potential limitations that warrant consideration when interpreting the findings. First, all patients underwent surgery at a single, high-volume cancer centre renowned for its adeptness in managing patients afflicted by severe comorbidities and offering a spectrum of oncological services. Each surgical procedure was conducted by seasoned thoracic surgeons who had transcended the learning curve and had completed 40–50 VATS lobectomies annually.

Second, it is worth noting that the number of cases included in the analysis might not be adequate for a comprehensive evaluation of survival rates. This insufficiency could introduce selection bias, which may undermine the generalizability of the study's conclusions. The potential bias introduced by collinearity among key variables in the logistic regression models. Notably, variables such as tumour stage, N status, and distance to hilum exhibited a degree of interdependence, raising concerns about the robustness of our models. While we implemented rigorous model selection techniques, including cross-validation, to mitigate the risk of overfitting, the inherent collinearity remains a challenge. Estimation of overall survival in the context of elderly cancer patients is complex and is potentially influenced by comorbidities. While our primary focus was on cancer-related endpoints, the study did not delve into a detailed examination of specific comorbidities and their impact on overall survival. But the prognostic power of CCI was validated and confirmed. Further propensity score matching or a prospective randomised trial is needed.

## Conclusion

In each case of lung cancer in patients above 75 years of age, an individualised approach is essential. The elevated mortality rate underscores the necessity for a comprehensive and meticulous assessment of risk factors. Preoperative intervention to ameliorate existing comorbidities should involve the active participation of anesthesiologists, intensive care physicians, cardiologists, and other relevant specialists, as dictated by the specific case requirements.

The convenience of a thorough discussion within the extended tumour board is of paramount importance in ensuring optimal patient care. Especially in the presence of negative prognostic factors such as stage IIIb, lymph node metastases, central tumour location, squamous cell histology, and the requirement for thoracotomy, the absolute risk of postoperative complications experiences a substantial increase. This underscores the criticality of precision and thorough planning in the management of elderly lung cancer patients.

### Clinical practice points

Increased mortality and morbidity rates in elderly patients with lung cancer require a careful and detailed assessment of risk factors and preoperative compensation for any existing comorbidity with the participation of an anesthesiologist, intensive care physician and cardiologist and other related specialists if necessary. In the presence of the identified risk factors, such as stage IIIb, lymph node metastases, central tumour location, squamous cell histology, and the necessity for thoracotomy, shared decision-making should be undertaken. This involves providing a thorough explanation of the associated risks, benefits, and alternative treatment modalities, including options such as SBRT or chemoimmunotherapy.

## Data Availability

The data underlying this article are available in the Dryad Digital Repository at https://doi.org/10.5061/dryad.kh1893289.
